# Supplementary material for: The Role of Thermokarst Lake Expansion in Altering the Microbial Community and Methane Cycling in Beiluhe Basin on Tibetan Plateau
Source: Microorganisms. 2022 Aug 10;10(8):1620. doi: 10.3390/microorganisms10081620 (PMC9412574; doi:10.3390/microorganisms10081620)
Supplement: Supplementary file 1 [file microorganisms-10-01620-s001.zip › microorganisms-1825792-supplementary.pdf]

Table S1 Information of domain-specific primers

Primer Name	Primer Sequence pairs with forward (F)	Primer Sequence pairs with reverse (R)
338F_806R	ACTCCTACGGGAGGCAGCAG	GGACTACHVGGGTWTCTAAT
524F10extF_Arch958RmodR	TGYCAGCCGCCGCGGTAA	YCCGGCGTTGAVTCCAATT
MLfF_MLrR	GGTGGTGTMGGATTCACACARTAYGCWACAGC	TTCATTGCRTAGTTWGGRTAGTT
A189F_mb661R	GGNGACTGGGACTTCTGG	CCGGMGCAACGTCYTTACC

Table S2 metadata

SampleID	Group	Date	Site	BarcodeSequence	LinkerPrimerSequence	ReversePrimer	Batch	Sequencing	Platform	Description
CE1	CE	2021/1/27	Sediment	ACGCTCGACA	ACTCCTACGGGAGGCAGCAG	GGACTACHVGGGTWTCTAAT	1	Majorbio	Miseq PE300	Shore replicate 1
CE2	CE	2021/1/27	Sediment	ATCAGACACG	ACTCCTACGGGAGGCAGCAG	GGACTACHVGGGTWTCTAAT	1	Majorbio	Miseq PE300	Shore replicate 2
CE3	CE	2021/1/27	Sediment	ATATCGCGAG	ACTCCTACGGGAGGCAGCAG	GGACTACHVGGGTWTCTAAT	1	Majorbio	Miseq PE300	Shore replicate 3
MC1	MC	2021/1/27	Sediment	CACGAGACAG	ACTCCTACGGGAGGCAGCAG	GGACTACHVGGGTWTCTAAT	1	Majorbio	Miseq PE300	Medium-shore replicate 1
MC2	MC	2021/1/27	Sediment	CTCGCGTGTC	ACTCCTACGGGAGGCAGCAG	GGACTACHVGGGTWTCTAAT	1	Majorbio	Miseq PE300	Medium-shore replicate 2
MC3	MC	2021/1/27	Sediment	TAGTATCAGC	ACTCCTACGGGAGGCAGCAG	GGACTACHVGGGTWTCTAAT	1	Majorbio	Miseq PE300	Medium-shore replicate 3
MS1	MS	2021/1/27	Sediment	TCTCTATGCG	ACTCCTACGGGAGGCAGCAG	GGACTACHVGGGTWTCTAAT	1	Majorbio	Miseq PE300	Medium-center replicate 1
MS2	MS	2021/1/27	Sediment	TACTGAGCTA	ACTCCTACGGGAGGCAGCAG	GGACTACHVGGGTWTCTAAT	1	Majorbio	Miseq PE300	Medium-center replicate 2
MS3	MS	2021/1/27	Sediment	CATAGTAGTG	ACTCCTACGGGAGGCAGCAG	GGACTACHVGGGTWTCTAAT	1	Majorbio	Miseq PE300	Medium-center replicate 3
SH1	SH	2021/1/27	Sediment	CGAGAGATAC	ACTCCTACGGGAGGCAGCAG	GGACTACHVGGGTWTCTAAT	1	Majorbio	Miseq PE300	Center replicate1
SH2	SH	2021/1/27	Sediment	ATACGACGTA	ACTCCTACGGGAGGCAGCAG	GGACTACHVGGGTWTCTAAT	1	Majorbio	Miseq PE300	Center replicate2
SH3	SH	2021/1/27	Sediment	TCACGTACTA	ACTCCTACGGGAGGCAGCAG	GGACTACHVGGGTWTCTAAT	1	Majorbio	Miseq PE300	Center replicate3
CE1	CE_B	2021/1/27	water	ACGCTCGACA	ACTCCTACGGGAGGCAGCAG	GGACTACHVGGGTWTCTAAT	1	Majorbio	Miseq PE300	Water Center replicate 1
CE2	CE_B	2021/1/27	water	ATCAGACACG	ACTCCTACGGGAGGCAGCAG	GGACTACHVGGGTWTCTAAT	1	Majorbio	Miseq PE300	Water Center replicate 2
CE3	CE_B	2021/1/27	water	ATATCGCGAG	ACTCCTACGGGAGGCAGCAG	GGACTACHVGGGTWTCTAAT	1	Majorbio	Miseq PE300	Water Center replicate 3
CE4	CE_M	2021/1/27	water	ACGCTCGACA	ACTCCTACGGGAGGCAGCAG	GGACTACHVGGGTWTCTAAT	1	Majorbio	Miseq PE300	Water Center replicate 4
CE5	CE_M	2021/1/27	water	ATCAGACACG	ACTCCTACGGGAGGCAGCAG	GGACTACHVGGGTWTCTAAT	1	Majorbio	Miseq PE300	Water Center replicate 5
CE6	CE_M	2021/1/27	water	ATATCGCGAG	ACTCCTACGGGAGGCAGCAG	GGACTACHVGGGTWTCTAAT	1	Majorbio	Miseq PE300	Water Center replicate 6
CE7	CE_T	2021/1/27	water	ACGCTCGACA	ACTCCTACGGGAGGCAGCAG	GGACTACHVGGGTWTCTAAT	1	Majorbio	Miseq PE300	Water Center replicate 7
CE8	CE_T	2021/1/27	water	ATCAGACACG	ACTCCTACGGGAGGCAGCAG	GGACTACHVGGGTWTCTAAT	1	Majorbio	Miseq PE300	Water Center replicate 8
CE9	CE_T	2021/1/27	water	ATATCGCGAG	ACTCCTACGGGAGGCAGCAG	GGACTACHVGGGTWTCTAAT	1	Majorbio	Miseq PE300	Water Center replicate 9
MC1	MC_B	2021/1/27	water	ACGCTCGACA	ACTCCTACGGGAGGCAGCAG	GGACTACHVGGGTWTCTAAT	1	Majorbio	Miseq PE300	Water Medium-shore replicate 1
MC2	MC_B	2021/1/27	water	ATCAGACACG	ACTCCTACGGGAGGCAGCAG	GGACTACHVGGGTWTCTAAT	1	Majorbio	Miseq PE300	Water Medium-shore replicate 2
MC3	MC_B	2021/1/27	water	ATATCGCGAG	ACTCCTACGGGAGGCAGCAG	GGACTACHVGGGTWTCTAAT	1	Majorbio	Miseq PE300	Water Medium-shore replicate 3
MC4	MC_M	2021/1/27	water	ACGCTCGACA	ACTCCTACGGGAGGCAGCAG	GGACTACHVGGGTWTCTAAT	1	Majorbio	Miseq PE300	Water Medium-shore replicate 4
MC5	MC_M	2021/1/27	water	ATCAGACACG	ACTCCTACGGGAGGCAGCAG	GGACTACHVGGGTWTCTAAT	1	Majorbio	Miseq PE300	Water Medium-shore replicate 5
MC6	MC_M	2021/1/27	water	ATATCGCGAG	ACTCCTACGGGAGGCAGCAG	GGACTACHVGGGTWTCTAAT	1	Majorbio	Miseq PE300	Water Medium-shore replicate 6
MC7	MC_T	2021/1/27	water	ACGCTCGACA	ACTCCTACGGGAGGCAGCAG	GGACTACHVGGGTWTCTAAT	1	Majorbio	Miseq PE300	Water Medium-shore replicate 7
MC8	MC_T	2021/1/27	water	ATCAGACACG	ACTCCTACGGGAGGCAGCAG	GGACTACHVGGGTWTCTAAT	1	Majorbio	Miseq PE300	Water Medium-shore replicate 8
MC9	MC_T	2021/1/27	water	ATATCGCGAG	ACTCCTACGGGAGGCAGCAG	GGACTACHVGGGTWTCTAAT	1	Majorbio	Miseq PE300	Water Medium-shore replicate 9
MS1	MS_B	2021/1/27	water	ACGCTCGACA	ACTCCTACGGGAGGCAGCAG	GGACTACHVGGGTWTCTAAT	1	Majorbio	Miseq PE300	Water Medium-center replicate 4
MS2	MS_B	2021/1/27	water	ATCAGACACG	ACTCCTACGGGAGGCAGCAG	GGACTACHVGGGTWTCTAAT	1	Majorbio	Miseq PE300	Water Medium-center replicate 5
MS3	MS_B	2021/1/27	water	ATATCGCGAG	ACTCCTACGGGAGGCAGCAG	GGACTACHVGGGTWTCTAAT	1	Majorbio	Miseq PE300	Water Medium-center replicate 6
MS4	MS_M	2021/1/27	water	ACGCTCGACA	ACTCCTACGGGAGGCAGCAG	GGACTACHVGGGTWTCTAAT	1	Majorbio	Miseq PE300	Water Medium-center replicate 4
MS5	MS_M	2021/1/27	water	ATCAGACACG	ACTCCTACGGGAGGCAGCAG	GGACTACHVGGGTWTCTAAT	1	Majorbio	Miseq PE300	Water Medium-center replicate 5
MS6	MS_M	2021/1/27	water	ATATCGCGAG	ACTCCTACGGGAGGCAGCAG	GGACTACHVGGGTWTCTAAT	1	Majorbio	Miseq PE300	Water Medium-center replicate 6

MS7	MS_T	2021/1/27	water	ACGCTCGACA	ACTCCTACGGGAGGCAGCAG	GGACTACHVGGGTWTCTAAT	1	Majorbio	Miseq PE300	Water Medium-center replicate 7
MS8	MS_T	2021/1/27	water	ATCAGACACG	ACTCCTACGGGAGGCAGCAG	GGACTACHVGGGTWTCTAAT	1	Majorbio	Miseq PE300	Water Medium-center replicate 8
MS9	MS_T	2021/1/27	water	ATATCGCGAG	ACTCCTACGGGAGGCAGCAG	GGACTACHVGGGTWTCTAAT	1	Majorbio	Miseq PE300	Water Medium-center replicate 9
SH1	SH_B	2021/1/27	water	ACGCTCGACA	ACTCCTACGGGAGGCAGCAG	GGACTACHVGGGTWTCTAAT	1	Majorbio	Miseq PE300	Water Shore replicate 1
SH2	SH_B	2021/1/27	water	ATCAGACACG	ACTCCTACGGGAGGCAGCAG	GGACTACHVGGGTWTCTAAT	1	Majorbio	Miseq PE300	Water Shore replicate 2
SH3	SH_B	2021/1/27	water	ATATCGCGAG	ACTCCTACGGGAGGCAGCAG	GGACTACHVGGGTWTCTAAT	1	Majorbio	Miseq PE300	Water Shore replicate 3
SH4	SH_M	2021/1/27	water	ACGCTCGACA	ACTCCTACGGGAGGCAGCAG	GGACTACHVGGGTWTCTAAT	1	Majorbio	Miseq PE300	Water Shore replicate 4
SH5	SH_M	2021/1/27	water	ATCAGACACG	ACTCCTACGGGAGGCAGCAG	GGACTACHVGGGTWTCTAAT	1	Majorbio	Miseq PE300	Water Shore replicate 5
SH6	SH_M	2021/1/27	water	ATATCGCGAG	ACTCCTACGGGAGGCAGCAG	GGACTACHVGGGTWTCTAAT	1	Majorbio	Miseq PE300	Water Shore replicate 6
SH7	SH_T	2021/1/27	water	ACGCTCGACA	ACTCCTACGGGAGGCAGCAG	GGACTACHVGGGTWTCTAAT	1	Majorbio	Miseq PE300	Water Shore replicate 7
SH8	SH_T	2021/1/27	water	ATCAGACACG	ACTCCTACGGGAGGCAGCAG	GGACTACHVGGGTWTCTAAT	1	Majorbio	Miseq PE300	Water Shore replicate 8
SH9	SH_T	2021/1/27	water	ATATCGCGAG	ACTCCTACGGGAGGCAGCAG	GGACTACHVGGGTWTCTAAT	1	Majorbio	Miseq PE300	Water Shore replicate 9

Table S3 The methane concentration at different water layer

**Layer**	**Depth（m）**	**water temp**	**sal**	**conc (uM)**
SH-T	0	0.65	0.1	0.20
SH-M	0.4	0.85	0.1	0.2
SH-B	0.8	1.4	0.2	0.19
MS-T	0	0.55	0	11.55
MS-M	0.5	1.2	0.1	11.3
MS-B	1	2.05	0.1	11.13
MC-T	0	0.55	0	88.78
MC-M	0.6	1.3	0.1	91.27
MC-B	1.2	1.85	0.2	81.03
CE-T	0	0.3	0	244.86
CE-M	0.75	1.55	0	197.69
CE-B	1.5	2.1	0.1	222.85

Table S4 In situ surface water environmental factors

Site	pH	O2	Water Temp	Cond
SH				
MS	10.24	80.9	0.4	840
MC	9.599	13.8	0.5	862
CE	9.205	22.8	0.1	689

Table S5 The oxygen profile of the thermokarst lake in summer

Site	Profile	O2 含量
P2	PM1(0-20)	9.670
P2	PM1(20-40)	9.665
P2	PM1(40-60)	9.666
P2	PM1(60-80)	9.637
P2	PM1(80-100)	9.410
P2	PM1(100-120)	8.383
P2	PM1(160)	8.241

P2	PM1(220)	8.179
P0	PM1(0-20)	9.054
P0	PM1(20-40)	9.055
P0	PM1(40-60)	9.078
P0	PM1(60-80)	9.022
P0	PM1(80-100)	8.822
P0	PM1(100-120)	7.672

Table S6 The physiochemical factors in water of the thermokarst lake

	Treat	DIC	DOC	TN	F	Cl	SO4	Na	K	Mg	Ca	PH	Cond
CE1	CE	90.44	102.55	2.135	128.07	91589.24	943.63	5186.25	754.17	7700.61	32912.77	8.94	1289
CE2	CE	90.44	102.55	2.135	128.07	91589.24	943.63	5186.25	754.17	7700.61	32912.77	8.94	1289
CE3	CE	90.44	102.55	2.135	128.07	91589.24	943.63	5186.25	754.17	7700.61	32912.77	8.94	1289
CE4	CE	92.86	103.35	2.477	394.34	75286.92	586.82	5479.51	784.6	7933.48	30011.89	9.02	1340
CE5	CE	92.86	103.35	2.477	394.34	75286.92	586.82	5479.51	784.6	7933.48	30011.89	9.02	1340
CE6	CE	92.86	103.35	2.477	394.34	75286.92	586.82	5479.51	784.6	7933.48	30011.89	9.02	1340
CE7	CE	90.32	104.25	2.234	409.63	120134.93	710.71	4996.48	751.71	7779.18	37566.01	9.13	1316
CE8	CE	90.32	104.25	2.234	409.63	120134.93	710.71	4996.48	751.71	7779.18	37566.01	9.13	1316
CE9	CE	90.32	104.25	2.234	409.63	120134.93	710.71	4996.48	751.71	7779.18	37566.01	9.13	1316
MC1	MC	61.77	82.83	1.8	192.17	85582.31	1839.13	4901.35	730.77	7719.55	37873.59	9.46	1009
MC2	MC	61.77	82.83	1.8	192.17	85582.31	1839.13	4901.35	730.77	7719.55	37873.59	9.46	1009
MC3	MC	61.77	82.83	1.8	192.17	85582.31	1839.13	4901.35	730.77	7719.55	37873.59	9.46	1009
MC4	MC	57.16	82.87	1.709	228.43	131184.19	2835.44	5057.64	728.88	7625.66	34314.83	9.49	1006
MC5	MC	57.16	82.87	1.709	228.43	131184.19	2835.44	5057.64	728.88	7625.66	34314.83	9.49	1006
MC6	MC	57.16	82.87	1.709	228.43	131184.19	2835.44	5057.64	728.88	7625.66	34314.83	9.49	1006
MC7	MC	56.59	85.32	1.831	193.38	85093.97	1736.38	5140.24	748.19	7941.94	30555.35	9.51	1022
MC8	MC	56.59	85.32	1.831	193.38	85093.97	1736.38	5140.24	748.19	7941.94	30555.35	9.51	1022
MC9	MC	56.59	85.32	1.831	193.38	85093.97	1736.38	5140.24	748.19	7941.94	30555.35	9.51	1022
MS1	MS	54.51	89.49	1.95	86.82	71322.24	2127.67	27369.06	1458.32	50090.23	29679.45	9.79	1084
MS2	MS	54.51	89.49	1.95	86.82	71322.24	2127.67	27369.06	1458.32	50090.23	29679.45	9.79	1084
MS3	MS	54.51	89.49	1.95	86.82	71322.24	2127.67	27369.06	1458.32	50090.23	29679.45	9.79	1084
MS4	MS	55.34	90.68	2.019	60.45	46715.17	1322.93	27853.26	1522.43	51012.55	30595.12	9.58	1082
MS5	MS	55.34	90.68	2.019	60.45	46715.17	1322.93	27853.26	1522.43	51012.55	30595.12	9.58	1082
MS6	MS	55.34	90.68	2.019	60.45	46715.17	1322.93	27853.26	1522.43	51012.55	30595.12	9.58	1082
MS7	MS	55.7	89.935	1.946	40.43	25940.3	781.49	27312.13	1499.14	50325.25	30270.89	9.64	1086
MS8	MS	55.7	89.935	1.946	40.43	25940.3	781.49	27312.13	1499.14	50325.25	30270.89	9.64	1086
MS9	MS	55.7	89.935	1.946	40.43	25940.3	781.49	27312.13	1499.14	50325.25	30270.89	9.64	1086
SH1	SH	52.2	89.99	2.179	81.25	76028.99	2557.54	21603.94	1397.23	71358.37	56548.78	9.72	742
SH2	SH	52.2	89.99	2.179	81.25	76028.99	2557.54	21603.94	1397.23	71358.37	56548.78	9.72	742
SH3	SH	52.2	89.99	2.179	81.25	76028.99	2557.54	21603.94	1397.23	71358.37	56548.78	9.72	742
SH4	SH	54.26	90.92	1.951	111.97	76285.79	2549.58	20265.36	1223.8	65440.24	41184.06	9.71	870
SH5	SH	54.26	90.92	1.951	111.97	76285.79	2549.58	20265.36	1223.8	65440.24	41184.06	9.71	870
SH6	SH	54.26	90.92	1.951	111.97	76285.79	2549.58	20265.36	1223.8	65440.24	41184.06	9.71	870
SH7	SH	53.86	90.59	2.023	92.26	84740.04	2814.33	21401.18	1347.74	68942.62	42311.19	9.68	1080

SH8	SH	53.86	90.59	2.023	92.26	84740.04	2814.33	21401.18	1347.74	68942.62	42311.19	9.68	1080
SH9	SH	53.86	90.59	2.023	92.26	84740.04	2814.33	21401.18	1347.74	68942.62	42311.19	9.68	1080

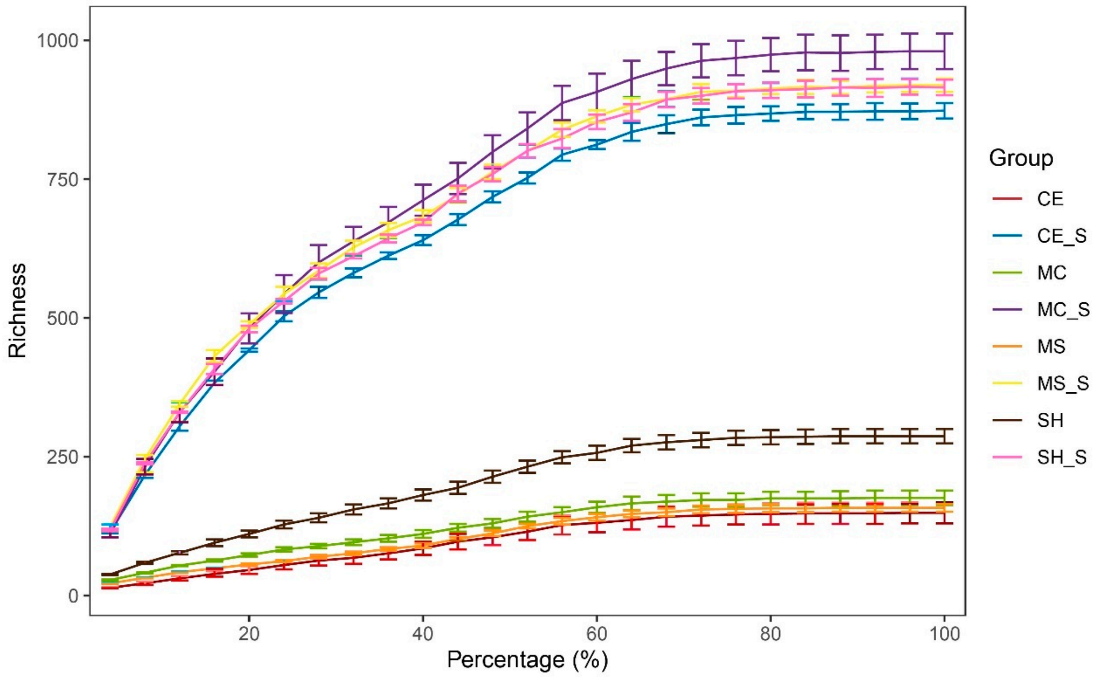

Figure S1 The rarefaction curves of bacteria in sediment and water. SH (SH in water), SH_S (SH in sediment), MS (MS in water), MS_S (MS in sediment), MC (MC in water), MC_S (MC in sediment), CE (CE in water) and CE_S (CE in sediment).

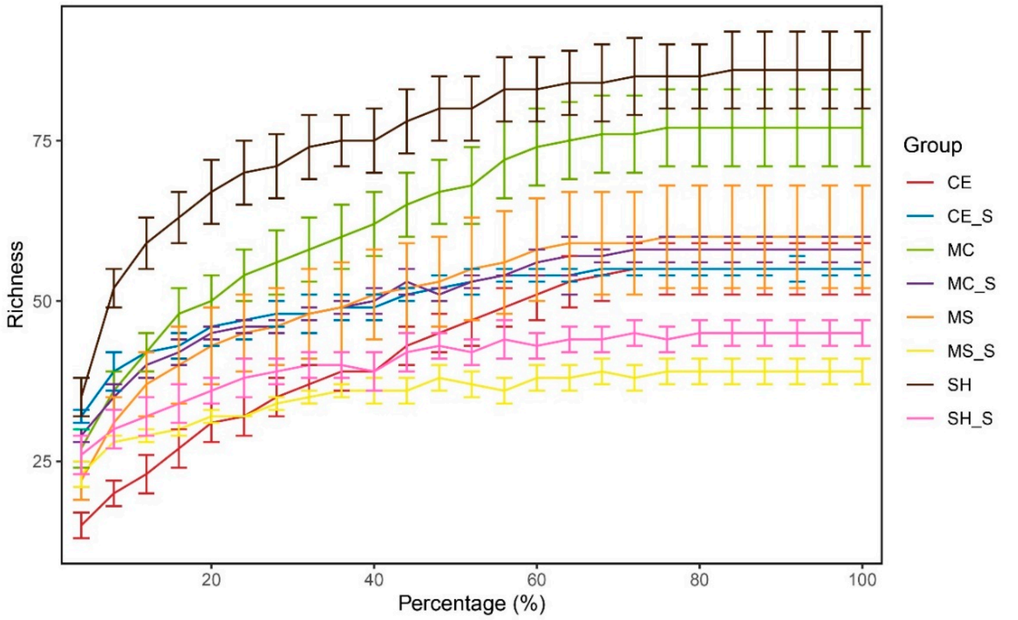

Figure S2 The rarefaction curves of archaea in sediment and water. SH (SH in water), SH_S (SH in sediment), MS (MS in water), MS_S (MS in sediment), MC (MC in water), MC_S (MC in sediment), CE (CE in water) and CE_S (CE in sediment).

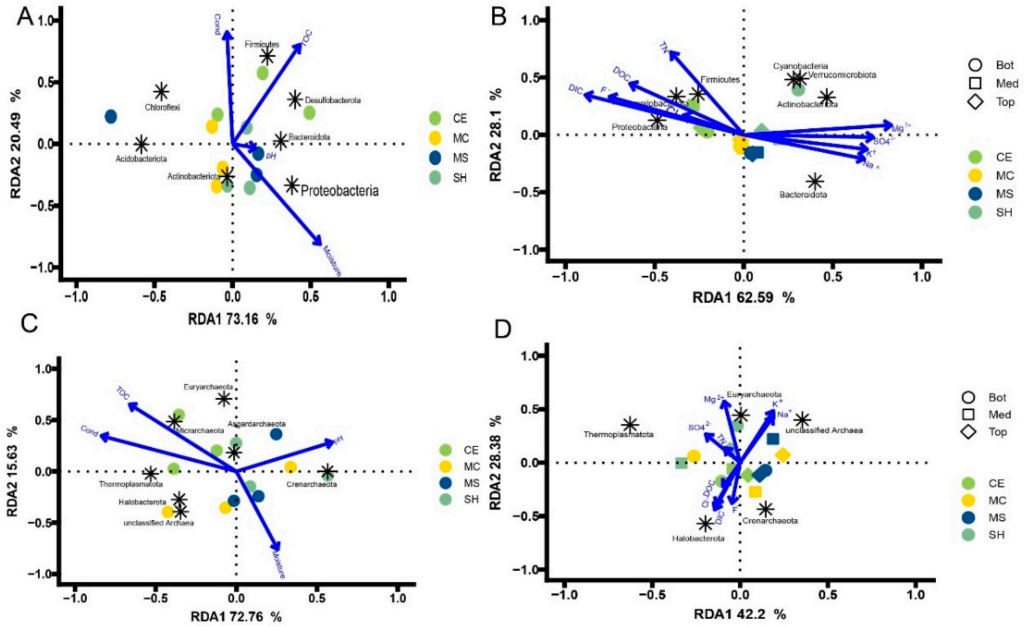

Figure S3 The relationships between microbial and environmental factors based on RDA. The relationships of (A) sediment and (B) water dominant bacterial phylum with environmental factors; the relationships of (C) sediment and (D) water dominant archaeal phylum with environmental factors. Four sampling points: SH, MS, MC, CE.

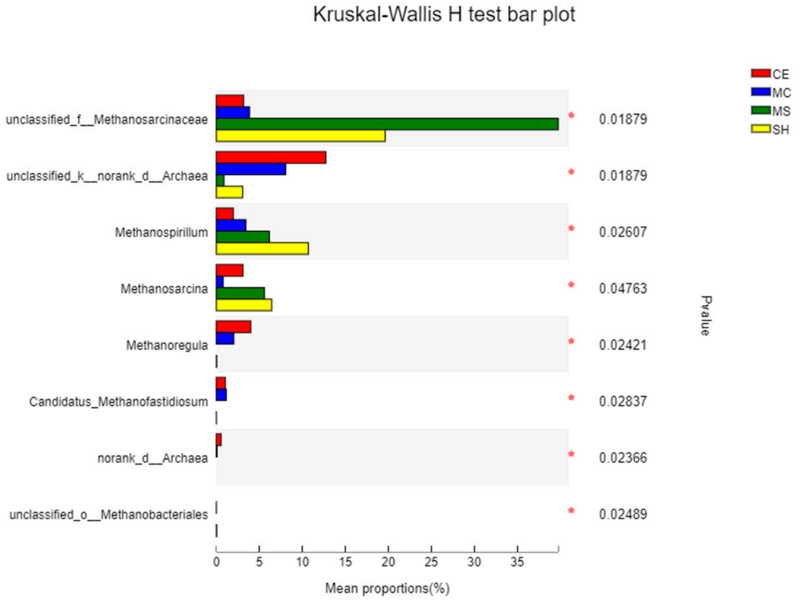

Figure S4 Microorganism involved in methane cycling with significant differences in sediment among points

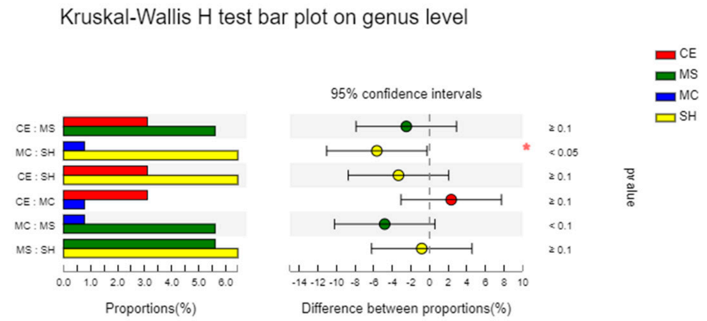

Figure S5 The relative abundance of *Methanosarcina* with significant differences among points

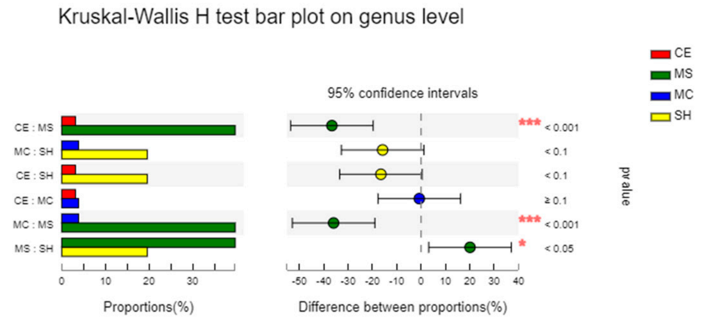

Figure S6 The relative abundance of Unclassified Methanosarcinaceae with significant differences among points

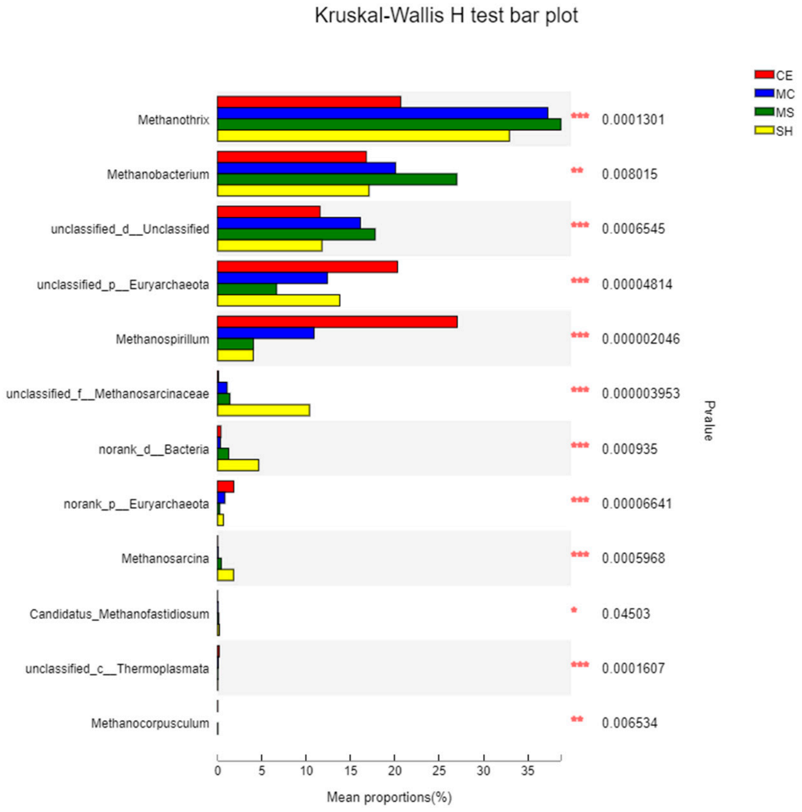

Figure S7 Microorganism involved in methane cycling with significant differences in water among points

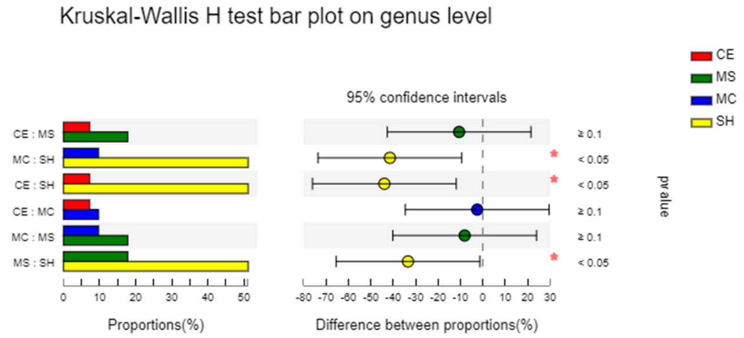

Figure S8 The relative abundance of *Methylobacter* with significant differences among points

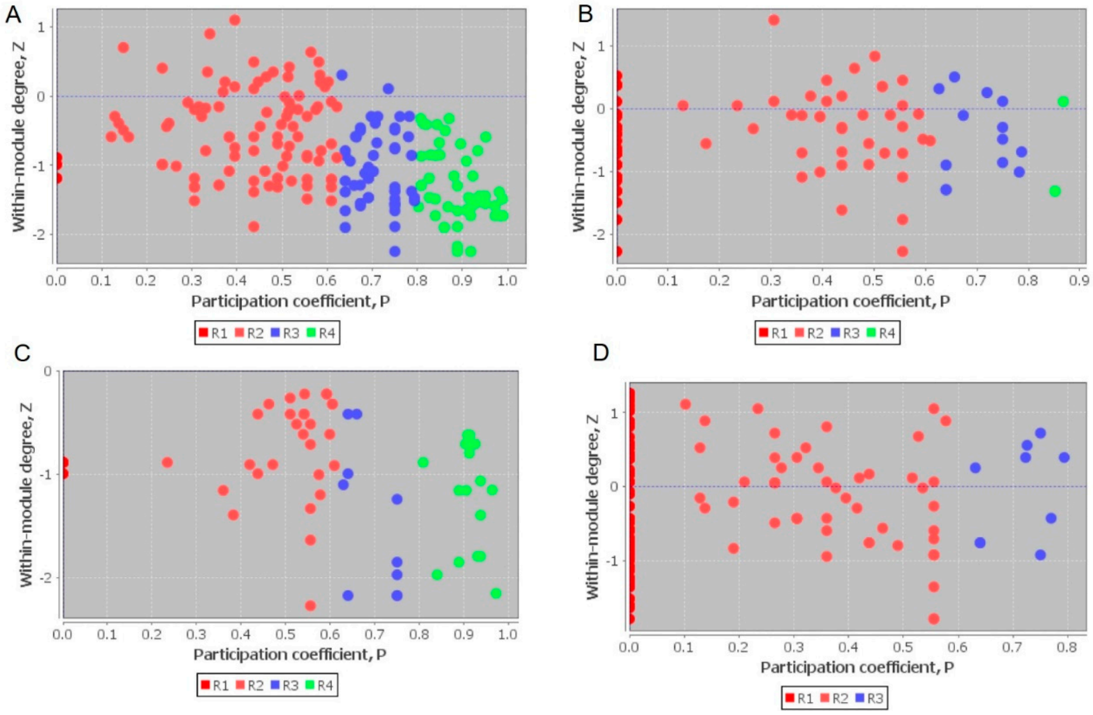

Figure S9 Topological roles of OTUs in the microbial co-occurrence networks as indicated by the *Zi-Pi* plot. (A) bacteria in sediment; (B) bacteria in water; (C) archaea in sediment; (D) archaea in water.
